# An Educational Problem

**Published:** 1910-07-09

**Authors:** 


### The Hospital

A Journal of

The Medical Sciences and Hospital Administration.

New Series. No. 178, Vol. VII. [No. 1250, Vol. XLVIII.] SATURDAY, JULY 9, 1910

### An Educational Problem.

The problems of medical education and the practical solutions evolved in the different medical schools of the United Kingdom are very numerous. There is perhaps none which is so formidable as that now presenting itself for solution in medical as well as other educational centres, the problem of the coloured student. It is not easy even to mention the question, let alone to discuss it, in language which shall be both sincere and incapable of misconstruction or offence; yet rather than ignore it it may be better to speak frankly and to deal as honestly with it as possible. The fact is that of quite recent years—that is, during the last decade—rapidly increasing numbers of British subjects, whose skins are darker than ours, have been enrolled as students in our medical schools. This fact inevitably causes some dislocation of the interior economy of any medical school in which these students are working. Rightly or wrongly, the Indian, the Egyptian, the West Indian negro, and other non-European races whose destiny has brought them within the British Empire, have been encouraged to seek Western culture and Western civilisation by coming to Britain for education. In most cases, naturally, it is only the wealthy who can afford this expensive training, though in a few cases religious and other charitable organisations have sent over boys of more straitened circumstances. Whether this encouragement has been unwise, whether trying to make East meet West is a mistake, either in principle or in detail, is a broad subject and a vastly important one, but it is not one that can be fitly thrashed out here. What have to be dealt with are the situation as it has arisen, and its probable developments.

Among the various arts and sciences of Occidental culture, medicine has been one of the last to be adopted by our Oriental visitors, and therefore to some extent it is possible to profit by the experience of other educational bodies in endeavouring to estimate the effect of their advent upon the medical schools. At the risk of offence, it may confidently be stated that an educational body which admits any considerable proportion of non-Europeans incurs, to a certainty, unpopularity and loss of prestige among the English. It may be due to an absurd insularity, but it is a fact that has to be faced and reckoned with. Experience has proved that this is the case with colleges at Oxford and Cambridge, and with every other place of liberal education at which the experiment has been tried. Without doubt the same result will be evident—nay, even is already evident—in the British medical schools; those which admit the particular nationalities now under consideration will decline in favour with the youth of this country. It may or may not be reprehensible, but the existence of the tendency is unquestionable. It may be asked how, if this is so, does any school open its doors to non-Europeans? The answer is simply that many are obliged to do so by the pinch of poverty. Among medical schools those of London have for some time felt the pinch of an undeserved poverty, and at the same time they offer an education at least as good as any others in the kingdom. Hence it is especially in London that the authorities may feel compelled, for financial reasons, to receive these entries. The results of this policy are manifold. In some ways it is at least potential of much good. If Orientals are to take up modern medicine and surgery, it is well that they should see it at its best; if they are to visit England, it is well that the great metropolis should be their selected residence. On the other hand, it may increase the already too great reluctance of English students to come to London; and charity, after all, begins at home. There is, moreover, an aspect of the matter which cannot be left out of account, and that is the patient's view. A great many men, and a still larger proportion of women, object very strongly to their wounds being dressed or their bodies palpated and otherwise examined by coloured students. In gynæcological and obstetric practice this objection is felt with additional force, and it is a grave obstacle in the way of complete medical training. There is no need to labour the point, but it is one so important that it must receive attention.

So much for the facts of the case. What of a remedy? It is, of course, of no use to tell the English student that he is uncharitable or narrow-minded; nor is it likely to be of any avail to explain to the hospital patient that his prejudices are indefensible. One plan that might be useful would be to attempt to divide the strangers as nearly equally as possible among the various schools so that there might be but a small percentage at each. This would tend to prevent the association of the non-Europeans in cliques and their severance from their fellow-students. Each would be more likely to be accepted on his own merits, and it would rest with the individual what his reception should be. A few years ago a Chinese student was admitted at a well-known medical school, after a preliminary career at one of the Universities. He was industrious, intelligent, and highly amiable, made many friends among the English students, and excited both affection and respect. In a word, he was a good fellow, and was speedily recognised as such. Similarly the Hindu, the Egyptian, the Malay, or any other representative of the non-Aryan subjects of the King, will each be taken at a fairly true valuation if he will apply himself seriously to his work in a British medical school. Character, after all, is the most important asset an education can bring out. Character is needed in the medical profession perhaps even more than in the Civil Service of an Asiatic or African dependency. It is one of the peculiarities of attendance on the sick that it develops character in a remarkable way; and it may be supposed that to pursue medical study in the lands of their birth will achieve this result as well as, if not better than, in the alien surroundings of Great Britain. If, therefore, a staff of excellent teachers can be secured—and this is simply a question of finance—it would seem both economical and advantageous all round to develop the schools of medicine, such as those that are already existing in India, until they can receive all those who are anxious to have a medical training. The Japanese have done this already, and there seems no reason why other Oriental peoples should not follow this example.

